# Building a laboratory at a Primarily Undergraduate Institution (PUI)

**DOI:** 10.1186/s12919-021-00208-5

**Published:** 2021-06-22

**Authors:** Caroline (Lina) Lund Dahlberg, Christina King-Smith, Blake Riggs

**Affiliations:** 1 Western Washington University, Bellingham, WA USA; 2 Saint Joseph’s University, Philadelphia, PA USA; 3 San Francisco State University, San Francisco, CA USA

**Keywords:** Primarily undergraduate institution (PUI), Laboratory, Teaching, Research, Start-up, Negotiation, Undergraduate

## Abstract

Scientists who are interested in building research programs at primarily-undergraduate institutions (PUIs) have unique considerations compared to colleagues at research-intensive (R1) institutions. Maintaining a research program at a PUI holds unique challenges that should be considered before prospective faculty go on the job market, as they negotiate a job offer, and after they begin a new position. In this article we describe some of the considerations that aspiring and newly hired faculty should keep in mind as they plan out how they will set up a laboratory as a new Principle Investigator (PI) at a PUI.

Anyone hoping to start a research program at a PUI should understand both the timeframe of interviews, job offers, and negotiations and the challenges and rewards of working with undergraduate researchers. Once a job is offered, candidates should be aware of the range of negotiable terms that can be part of a start-up package. Space and equipment considerations are also important, and making the most of shared spaces, existing infrastructure, and deals can extend the purchasing power of start-up funds as a new PIs builds their lab. PUIs’ focus on undergraduate education and mentorship leads to important opportunities for collaboration, funding, and bringing research projects directly into undergraduate teaching laboratories.

A major focus of any new laboratory leader must be on building a productive, equitable, and supportive laboratory community. Equitable onboarding, mentorship plans, and formalized expectations, can all help build a productive and sustainable laboratory research program. However, important considerations about safety, inclusion, student schedules, and a PI’s own professional commitments are also extremely important concerns when working with undergraduates in research. A successful research program at a PUI will bring students into meaningful scientific inquiry and requires insights and skills that are often not the focus of scientific training. This article aims to describe the scope of setting up a new laboratory as a way to alleviate some of the burden that new and prospective faculty often feel.

## Background

Participation in research has important impacts on students, including gains in science identity and retention in biological science [1–3]. Therefore, it is critical that laboratory research at PUIs be centered around faculty with the interest, motivation, and resources to effectively train, mentor, and support their student researchers. Scientists who are interested in leading undergraduate research laboratories often have extensive training in their own research field but not in how to apply for, equip, or start up a laboratory of their own [4–7].

There is a common misconception that research at a PUI is easier than at an R1 institution, however there are many challenges that make leading a PUI research program more difficult. These include anticipating what kinds of research are compatible with high teaching and service loads and designing a research program that engages undergraduates. Starting a research program at a PUI will likely involve a shift in expectations but that does not mean that research has to halt or become less interesting or less rigorous. With a reasonable research plan and the right equipment, undergraduate-focused research programs are productive and stimulating for both the students and the PI.

Planning a laboratory at a PUI should start even before applying and interviewing for a position [8–10]. Institutions generally select a list of competitive candidates, screen them via phone or video interview, invite two or three top candidates for in-person interviews, and make a job offer (for an example timeline see reference 6). It is important that candidates prepare for each of these steps though the lens of developing a research program. One approach is to consider the following questions, which are similar to those that might be used in a phone or in-person interview.

- What does your ideal start up package look like?
- What will your laboratory *need* for your research to be successful?
- What elements from your previous laboratory experiences would you like to adopt? Which would you hope to avoid?
- Who (and how many people) would be in your ideal laboratory?
- How will you protect your time and achieve your goals?

Having clear answers or several workable options for these can provide a good platform from which to engage with more detailed questions. In this article, we have provided a series of ideas, hints, and advice that correspond to each of these broader questions.

This set of questions and the descriptions below was originally presented as part of the American Society for Cell Biology’s Advancing Career Transitions (ACT) workshop series for fellows funded by an NIH IPERT grant. A focus of this grant is to support a more diverse science workforce by providing professional development to early career scientists who are interested in pursuing faculty positions.

## What is a start-up package?

A start-up package is the combined funding and non-monetary portions that accompany a job offer. Start-up packages usually include 1) funds to equip and support a new research laboratory and research program, 2) teaching load progression, 3) service load progression, 4) fringe and other benefits, and 5) start time. Both an institution and a job candidate can negotiate the terms of a job offer, including the start-up package.

Start-up packages are important because they set up a new investigator for success. In addition, new faculty need to think about how they will best be able to use it. The final section of this article provides some suggestions and resources to look for so that your research goals are attainable.

### Space and equipment needs and purchases

It is thrilling to walk into your new lab space, but it can be overwhelming to think about how to equip it. Often job applicants will be asked to provide a list of equipment needs during interviews (see accompanying article, King-Smith, et al.). One strategy for developing this list is to map out a grant or manuscript and plan backwards for the necessary resources to gather the data. It also is a good idea to earmark a percentage of extra spending in case a research program changes direction, requires novel collaborations, or runs into unforeseen challenges. PUIs that require evidence of research productivity for reappointment, tenure, and promotion generally have different expectations for publication, external funding and student authorship, compared to R1 institutions. If getting a grant or publishing research with students is particularly important for tenure and promotion, then equipping your lab to achieve those goals should help guide your start-up spending.

Many departments have shared equipment and core facilities and it is important to find out what limits and costs there are for their use, upkeep, or adaptations. Remember that simply having the funds to get a large piece of equipment does not guarantee that there will be space for it once it arrives. If a lab space itself cannot hold the equipment be prepared to work with space committees to find an appropriate place for it. In addition, costs for service contracts, which can be significant, may not be covered in the cost of equipment, but may be critical for some purchases. Communicate with department members to see there are ways to share space or costs for equipment.

Spending start-up funds is often an exercise in comparison shopping. If a research group that you have worked in previously is willing to share their purchasing lists, using those as a starting point can save a lot of time. Sales representatives can help identify good values, provide bundled prices, or find educational discounts. On the other hand, new lab and new grant deals are often geared toward R1 labs and are generally not efficient uses of a PUI startup, especially if they are one-time only. Refurbished or re-sale equipment offer good deals, although it should be obvious that resale sites require the buyer to beware. Professional or science community listserves and word of mouth are good sources of information about established laboratories or biotech companies that are closing or upgrading, which are great sources of used equipment. Be prepared to transport your prizes and to take precautions regarding safety or contamination. Also be aware that institutions barcode and catalog most equipment, so you might not be *allowed* to take something, even if a PI offers it.

### Finding support, funding, and collaborations

PUIs include a wide variety of employees who support undergraduate education and research. While faculty will clearly be important resources for advice and intramural research and teaching collaborations, custodial, administrative, research and sponsored programs (grants), and purchasing staff and stockroom and laboratory support personnel can be truly invaluable. Getting to know the names and faces of the staff who facilitate your work will make working with them easier and more fun. They are as dedicated as faculty to the success of students and research programs and they will smooth the transition from new to established PI.

PUIs provide important opportunities for research to be included in coursework and part of a research program can often fit into existing coursework, or into new courses that include student-friendly experiments or analyses. Bringing your research into a course is not simple, but it can provide students who otherwise wouldn’t get a chance to join a research lab with research experience. Additionally, PUIs sometimes provide financial support for reagents and equipment that are used as part of a course. For more information on Course-based Undergraduate Research Experiences (CUREs) see references [11–13].

It is important to strategize about whether your efforts should focus on publishing versus getting grant funding. If an institution does not prioritize a PI’s ability to bring in grants, it could be wise to focus on publishing. Conversely if grant funding is more valued, it is worth working towards grant funding immediately. It is worth noting that grant funding does facilitate publishing indirectly by funding publication costs, summer research costs and salary, teaching buy-outs, and support personnel such as technicians and post-docs. Several federal and local funding opportunities exist for PUIs. Both the NSF and NIH have PUI-specific funding mechanisms (see accompanying article, King-Smith., et al. and [14] for examples and contacts) and the teaching focus at PUIs can be highlighted as a Broader Impact for NSF grants. Discuss your grant ideas with the appropriate NSF or NIH program officer and obtain feedback throughout the grant writing process to help guide your thinking and writing.

Regardless of the agency, grants must be well-written and accessible. Writing workshops, working groups with other new faculty, and soliciting funding advice and feedback from senior faculty can also increase the chances of successful grant funding. Workbooks are available from the Grant Writers’ Seminars and Workshops which provide easy-to-follow framework for proposals [15]. Grant-writing is an iterative process so submitting a grant early on ensures that you will have time to revise and resubmit giving you better chance of being funded before tenure. In addition, collaborations that include undergraduate-friendly projects can move projects forward towards new funding. Some examples of collaborations include:

- Inter- or multi-disciplinary research with other labs at your institution.
- Collaborations with R1 labs, which are often happy to help support smaller scale laboratories.
- Community colleges serve the majority of undergraduate students in the US, and their faculty are often interested in partnerships.
- Connecting to networks of Hispanic Serving Institutions (HSIs), Historically Black Colleges and Universities (HBCUs) and other Minority Serving Institutions (MSIs) strengthen and broaden the impact of a grant.

If you are bringing funding from another position be aware that indirect costs may be different, and that previously budgeted costs could differ significantly. If funding can be used to “buy out” of teaching, find out how those costs are calculated so that you can make informed decisions about what courses or academic terms you will replace with support from research funding.

### Design a strategy for on-boarding new lab members and building a team

Building an inclusive, equitable laboratory community is crucial, and it will not happen by accident. Clear mentoring philosophies, mentoring action plans, and mentoring compacts can help delineate expectations for all members of a laboratory and will help build a productive rapport between PIs and students (See accompanying article, Diggs-Andrews et al. and [16]). Faculty should be aware of the inequities that can be bred and deepened in a laboratory group and should educate themselves on how to avoid perpetuating existing inequity [17–21].

When planning a laboratory around undergraduate researchers, it is crucial to consider the time that it takes to teach, mentor, and troubleshoot with scientists who are at the very beginning of their career. As they learn, students will make mistakes and they will require both oversight and encouragement. Our advice is to start small (for example, two students working together). In addition, consider the level experience students will have. There are advantages to both inexperienced or introductory-level students and more advanced students, and a mix of both is often a great way to ensure that a lab remains productive as students graduate or leave the lab group. This “terraced” effect keeps talent and expertise consistent in the lab group. Hiring costs for employees such as technicians are very high and are not an efficient use of start-up funds. At PUIs that also train graduate students, it is important that graduate students share the goals of supporting and collaborating with undergraduates so that their expertise benefits all members of the laboratory. Overall, while space constraints will dictate the size of your lab to some extent, designing a supportive, inclusive laboratory community will be more about the quality of the experiences than the number of lab members. Students help spread the reputation of a laboratory, which can help attract the next generation of students to carry it forward.

While it is tempting to accept all interested students into a new laboratory, time and space are limited. Understanding how potential student researchers react to challenges, motivation and commitment will help narrow the field. Asking applicants to talk about challenges that they have encountered in classes or outside of lab, sending them literature to read for a future discussion, or encouraging them to describe their interest in popular science or course-work can give a clear impression of what a student will bring to a laboratory group. Another strategy is to ask students to commit to volunteering for a quarter or semester prior to making a longer commitment to the lab. As you make decisions and invitations, be mindful of your own biases: student researchers are joining a community. They should be encouraged to bring their unique perspective and style to the laboratory.

Good mentorship is crucial to maintaining a successful laboratory. High quality mentoring is most often built on strong, two-way communication that relieves some of the hierarchical pressures of scientific research. Some important resources for faculty mentors are the National Academies of Science and Engineering report and online guide on the Science of Effective Mentoring in STEM and Entering Mentoring along with the companion for undergraduates, Entering Research [16, 22–25]. Students can also mentor each other in pairs or groups for peer or near-peer mentoring. This style of mentoring can both provide students with agency in solving problems and draw on advanced students’ experiences to provide insights to newer students. Peer mentorship does not remove a faculty member from the mentorship relationship with any given student, and it is important to remember that a PI has power and responsibility that students do not [16].

Lab meetings are good ways to build community in a lab and keep a consistency going when things can be inconsistent in students’ schedules. Because undergraduate students have packed schedules, and can be inexpert in reading primary literature, journal clubs are terrific ways to bring all members of the group into the conversation, even if research-driven lab meetings aren’t always possible. Active learning strategies such as jigsaws, just-in-time teaching, and exit slips [26, 27] and resources and protocols such as C.R.E.A.T.E. [28, 29], Figure Facts [30], and Genetics Primers [31] can be useful frameworks for discussions of papers early in an academic year, or when students could use more guidance. Research presentations are extremely important for staying on top of students’ current findings and for pinpointing potential problems early. Useful scientific thinking such as formulating new hypotheses, troubleshooting, understanding the value of negative results, and collaboration are often some of the highlights of undergraduate research meetings.

A major source of laboratory community can also come from laboratory alumni. Therefore, it is worth thinking about how you will support students as they graduate from your laboratory. Your students will probably directly benefit from your own network and laboratories that you are connected with that could take your students as techs or grad students. The future success of your laboratory students is a metric that you can highlight for tenure and promotion and following the successes and careers of former students is extremely rewarding on a personal level.

### Considerations for working with undergraduates

Student research can take several forms and will require different time commitments. Students can have independent, but interrelated projects, they can work as a team on the same project, or work side-by-side with their PI. Students often gain valuable experience and confidence from being able to repeat procedures, and this can also reduce the need for your constant, direct supervision. As the lab builds, consider asking more senior students to train newer students so that they can act as peer mentors within the lab group.

Because different students will have different time availability for research, have clear expectations for the time commitment for undergraduate and graduate student researchers, research volunteers, and students doing research for course credit. One strategy is to have students post their schedules in the lab so that everyone can know who they can expect to see around. Some students may also benefit from having regular (weekly or monthly) goals, especially if they are earning course credit or a salary. Be flexible, as student interests may change, and crises can arise without warning. If a student needs to take time away from the lab, you will need to have a realistic discussion with them about what returning will look like, or the fact that you may assign their project to another student.

Student authorship and ownership on their own project is crucial to students’ development of their own sense of science identity and belonging. However, their work will reflect on the PI (you!), so make sure that their writing and presentations meet high standards. Concept mapping, outlines, annotated bibliographies, and peer-reviews can help frame writing projects, and there is a growing body of literature on their effectiveness in student learning [32–35]. Set reasonable deadlines that will allow you to work with students on reviews, revisions, flaws in logic, and style. Jan Pechenik’s A Short Guide to Writing About Biology is a straightforward primer for writing guidance [36].

While many labs rely on undergraduates who receive course credit or work as unpaid research volunteers, this can inherently support inequities because many students need to spend time out of class working to support themselves. Consider hiring research students using work-study support or start-up funds or hiring work-study students to help maintain your lab. Encourage students to apply for internal support for their hourly work, or purchase of reagents.

Many faculty at PUIs get much of their research done during the summer, so having talented undergraduates in the lab during the summer months can be very useful. Summers can also be great for training in newer and/or local students ahead of next academic year’s research. In addition, summer internships or fellowships at other institutions are excellent training for students, who will return in the fall with new perspectives and possibly new skills.

Finally, follow the institution’s policy for safety. If students can be in the lab after hours, or without supervision, build a policy for safe lab practice, for example a buddy system paired with a phone tree.

### Negotiating a start-up package

Having considered the topics above, it is important that you negotiate the strongest possible start-up package to support your research program. Negotiating a start-up package is almost always stressful but preparation can make this first step in starting a lab a bit easier. Having multiple job offers is extremely advantageous when it comes to negotiations, but regardless once you have a job offer, search salary databases and course catalogs to anticipate salary (ies) and teaching load(s). Asking current faculty for advice can also be very helpful. While a dean or provost may be hoping to save money on a new hire, departments are working to provide a new faculty member with the best-possible start to a new career.

A job offer will usually include an initial suggestion of start-up package, and this is certainly something to negotiate! It is valuable to have an idea of what comparable start-up packages are for other PUIs. It is appropriate to ask colleagues at other PUIs about their start-up packages; knowing how similar institutions fund their faculty is helpful for determining how flexible–or inflexible—to be during a negation. In section 1, we outlined portions of a startup package; below we describe some of these in more detail.

1. Transparency and honesty will help ensure that you have the funding and space that you need to start your new laboratory. At the same time, startup negotiations should reference full price new equipment: once you get into your new lab you can spend your money “differently” than you planned, including to pay student researchers. In the face of a start-up budget with little or no room for negotiation, a piece of equipment that will benefit many members of the department may be more attainable than earmarked funding for one new faculty member.
2. Teaching will take up more time that you expect so if it is possible, ask to repeat courses, and/or ensure that you can bring research into coursework are important considerations. In some cases, mentoring and doing research with undergraduates may count as part of your course load, and this should be clarified as part of your negotiation.
3. You may be able to negotiate regular, continued funding once startup money runs out, especially as a way to support research once start up is over. Small sums ($1000–$2000 per year) could help fund a small research project in the absence of external grant funding.
4. While service loads for new faculty often start low, they should be discussed explicitly with the department chair. It can be helpful to be forthright about what you would be most interested in so that your passions are fed through some of the service that you do.
5. Benefits including childcare, office space or location, parking permissions, extensions on when start-up funds must be spent, renovations, or equipment refurbishment can all be negotiated as part of the start-up package.
6. Start time: Institutions may be flexible regarding when a position must begin, other than the beginning of the academic year.

### Protecting your time so you can achieve your research and laboratory goals

New faculty often feel pressure to prove themselves as a dynamic and independent, which can lead to overpromising and burnout. In addition, building and maintaining a laboratory requires time management that is particularly difficult when starting a new faculty position. The following ideas can help establish and maintain balance so that a research program can thrive:

- Form or find a network of mentors. The National Mentoring Resource Network (NMRN, www.nrmnet.net), the National Center for Faculty Development and Diversity (NCFDD, www.facultydiversity.org) and professional organizations can provide connections and guidance. Mentors and peers can help with many of the points below.
- Find ways to take on reasonable service commitments, while honoring your passions for teaching, research, outreach, etc. Discuss your interests and limits with your department chair or faculty mentor. Contact institutional outreach offices and faculty or collaborators with similar interests to form partnerships for service that fulfills your obligations and is personally fulfilling.
- Set boundaries and goals by talking with colleagues, peers, and other mentors about what the teaching and service assignments from previous years were, and if they were achievable. You can also find out more about the realistic expectations for funding and publishing from lists of grants or publications from your institution or department.
- Find and foster collaborations that will serve your goals through faculty research networks on campus and via professional networks in your field.
- Set times in a calendar for office hours, teaching preparation, lab work and mentoring students, so that you ensure that service and other obligations do not take up more time than necessary.
- Embrace the “good enough” for first-drafts, first-round course-planning, and initial versions of other materials. Seek feedback from peers, trusted colleagues in your field, and teaching and learning centers at your institution for revisions.

## Conclusion

Successfully running a laboratory at a PUI requires a special focus on student success that bridges teaching and research. The learning curve for this is often steep, and it is not unusual for new faculty to feel as though they have made no scientific progress in their first year. Rest assured that this is normal. Establishing a balance between lab work, writing, mentoring, and teaching is vital, and will take time, but it is important to think about what that balance might look like as you set up, equip, and populate your new laboratory. By setting yourself up for success, you will be better able to help your students achieve success, as well.

## Data Availability

Not applicable.

## Abbreviations

PI: Principle investigator
PUI: Primarily undergraduate institution
R1: “Research 1” or research-intensive institution
